# Dynamic changes of neutrophil-to-lymphocyte ratio in brain-dead donors and delayed graft function in kidney transplant recipients

**DOI:** 10.1080/0886022X.2022.2141646

**Published:** 2022-11-08

**Authors:** Yongfang Zhang, Rumin Liu, Xiaolin Zhao, Zhiyu Ou, Shengnan Wang, Dongmei Wang, Kaibin Huang, Suyue Pan, Yongming Wu

**Affiliations:** a Department of Neurology, Nanfang Hospital, Southern Medical University, Guangzhou, China; b Department of Kidney Transplantation, Nanfang Hospital, Southern Medical University, Guangzhou, China

**Keywords:** Neutrophil-to-lymphocyte ratio, brain-dead donor, acute kidney injury, delayed graft function, kidney transplantation

## Abstract

**Objectives:**

Neutrophil-to-lymphocyte ratio (NLR) is a simple parameter implying the inflammatory status. We aimed to explore the association of brain-dead donor NLR change with delayed graft function (DGF) in kidney transplant recipients.

**Methods:**

We retrospectively analyzed the data on 102 adult brain-dead donors and their corresponding 199 kidney transplant recipients (2018 − 2021). We calculated ΔNLR by subtracting the NLR before evaluating brain death from the preoperative NLR. Increasing donor NLR was defined as ΔNLR > 0.

**Results:**

Forty-four (22%) recipients developed DGF after transplantation. Increasing donor NLR was significantly associated with the development of DGF in recipients (OR 2.8, 95% CI 1.2 − 6.6; *p* = .018), and remained significant (OR 2.6, 95% CI 1.0 − 6.4; *p* = .040) after adjustment of confounders including BMI, hypertension, diabetes, and the occurrence of cardiac arrest. When acute kidney injury (AKI) was included in the multivariable analysis, increasing donor NLR lost its independent correlation with DGF, while AKI remained an independent risk factor of recipient DGF (OR 4.5, 95% CI 2.7 − 7.6; *p* < .001). The area under the curve of combined increasing NLR and AKI in donors (0.873) for predicting DGF was superior to increasing donor NLR (0.625, *p* = .015) and AKI alone (0.859, *p* < .001).

**Conclusions:**

Dynamic changes of donor NLR are promising in predicting post-transplant DGF. It will assist clinicians in the early recognition and management of renal graft dysfunction. Validation of this new biomarker in a large study is needed.

## Introduction

Kidney transplantation is the therapeutic option for patients with end-stage renal failure. Complications after transplantation influence graft function and even contribute to graft loss, which lay a great social and economic burden on the healthcare system [1]. Delayed graft function (DGF) in kidney transplant recipients is not uncommon and the incidence is reported from 2% to 50% [2]. Currently, the most accepted definition of DGF is the requirement of at least one dialysis during the first week following transplantation [1–3]. The development of DGF prolongs the hospital’s stay and contributes to a 40% chance of graft failure in the first year after transplantation [4,5]. Accumulating evidence indicates that ischemia/reperfusion injury plays a vital role in the mechanism underlying DGF [2, 6–8]. A release of free radicals and the damage on vascular endothelium following ischemia/reperfusion injury aggravate the inflammatory response, causing deterioration of organ function [9].

The neutrophil-to-lymphocyte ratio (NLR) is an inexpensive, easy-obtained, widely available parameter to evaluate inflammation status. Previous literature suggested the predictive role of NLR in outcomes of many diseases including stroke, cardiovascular disease, and chronic kidney disease [10–12]. Dynamic changes of NLR have been reported to be related to acute kidney injury (AKI) [13–15]. Parlar et al. showed that increased NLR was related to postoperative AKI in cardiovascular surgery patients [14]. In addition, the association between NLR in kidney transplant recipients and graft outcome (e.g., incidence of DGF and acute rejection) has been investigated. They concluded that NLR value in recipients reflects the potential inflammatory state [16–18]. However, the optimal cut-off value of NLR remains inconsistent due to differences in the time point of blood samples, the calculating method, and clinical characteristics of the patient cohorts.

Organs procured from brain-dead donors is the main source used in transplantation [19]. However, graft prognosis is impaired by brain-dead related events. One of the important pathophysiology of renal graft dysfunction is the profound inflammatory response in brain-dead donors [20]. Before organ procurement, the inflammatory status in brain-dead donors is dynamically changing. Thus, the change of NLR calculated in donors might indicate various inflammatory stage. To date, the association between brain-dead donor NLR change and DGF in renal transplant recipients has not been reported. In this study, we aimed to analyze the correlation between brain-dead donor NLR change and the occurrence of DGF in renal transplant recipients.

## Methods

### Study design and population

Adult brain-dead patients who donated their kidneys in our institution between January 2018 and December 2021 were enrolled. Data of brain-dead donors and their corresponding recipients were reviewed from the electronic records. The information of their corresponding recipients was provided from the local Organ Procurement Organization (OPO) and the department of transplantation in our institution.

### Definitions of variables and outcomes

NLR value was defined as the ratio of absolute neutrophil count and absolute lymphocyte count in the peripheral blood. NLR was collected from the blood drawn at two periods: within 24 h before evaluating brain death (aNLR) and within 6 h before organ procurement (pNLR). The dynamic change of NLR (ΔNLR) was calculated by subtracting the NLR before evaluating brain death from the preoperative NLR (ΔNLR = pNLR – aNLR), and increasing donor NLR referred to ΔNLR > 0.

Expanded criteria donors (ECDs) were defined as age ≥ 60 years, or age 50–59 years with two of the following: history of hypertension, serum creatinine level ≥ 133 μmol/l (1.5 mg/dl), or cerebrovascular disease as a cause of death [8]. According to AKI network criteria based on serum creatinine from admission to the terminal (irrespective of time between measurements and of urine output cutoffs), AKI was divided into three stages: stage 1, increase in serum creatinine ≥0.3 mg/dl (≥26.4 μmol/l) or 1.5–2 fold increase; stage 2, 2–3 fold increase in serum creatinine; and stage 3, >3 fold increase in serum creatinine, or serum creatinine ≥4.0 mg/dl (≥354 μmol/l) with an acute rise of at least 0.5 mg/dl (44 μmol/l) [21]. Recipients who had a serum creatinine level >400 μmol/l after post-transplant 7 days and/or needed hemodialysis during the first week after transplantation were diagnosed with DGF [22,23]. Acute rejection was assessed *via* Banff criteria by allograft biopsy [24].

No prisoners or non-consenting donors were enrolled. The study was performed adhere to the principles of the Declaration of Helsinki. The Ethics Committee of Nanfang Hospital approved this research (NFEC-2021-410). Procurement of kidneys from brain-dead donors was approved by the Human Organ Transplantation and Ethics Committee of local institution. Organs were obtained by the Organ Procurement Organization of the local hospital and allocated by the China Organ Transplant Response System.

### Statistical analysis

Statistical analysis was performed on SPSS for Windows (version 25.0, IBM Corp., Armonk, NY). *P* value < .05 was considered statistically significant. Quantitative variables were expressed as median and 25th–75th interquartile ranges (IQRs), while categorical variables were expressed as number (percentages). Comparisons were made using Mann–Whitney test for continuous variables. The χ^2^ test or Fisher exact test was used for categorical variables. To account for the cluster effect of paired kidneys from the same donor, we used generalized estimating equations (GEEs) to perform multivariable analysis [25,26]. In the multivariate model, variables with a value of *p* < .05 by univariate analysis were included. The value of increasing donor NLR in predicting graft DGF was evaluated by receiver operating characteristic (ROC) curve.

## Results

**Figure 1.**
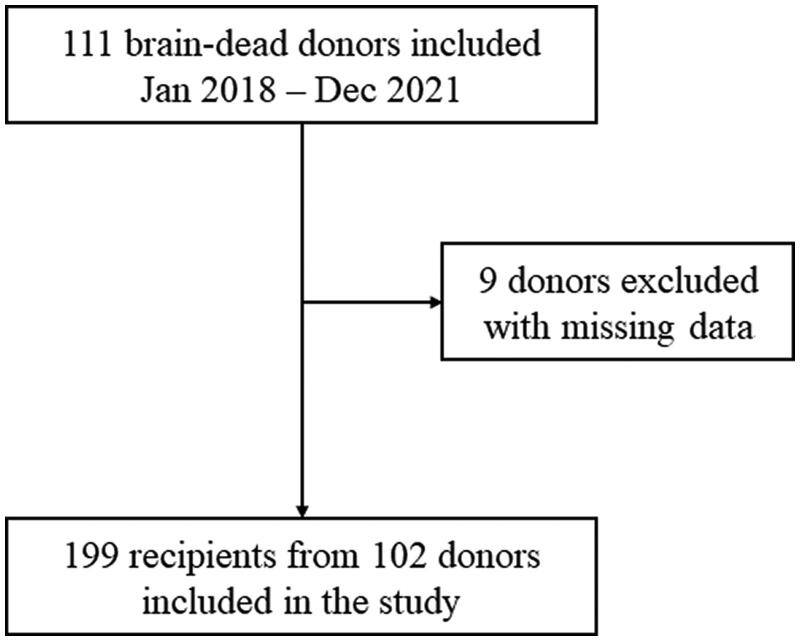
Flowchart of the inclusion.

During the study period, a total of 102 adult brain-dead donors and their corresponding 199 kidney transplant recipients were included (Figure 1). Both kidneys were donated in most donors. In five donors, only one kidney was transplanted. The incidence of DGF in the recipients was 22% (44/199). The rate of acute rejection in the study was 6%. The median age for donors was 47 (33–54) years, and 18 (18%) donors were ECDs. Donor-related factors including high BMI, history of hypertension or diabetes, occurrence of cardiac arrest, and AKI were statistically different between the DGF and non-DGF group (*p* < .05) (Table 1). Recipient characteristics are summarized in Table 2. There were insignificant differences in the clinical characteristics of recipients between two groups (*p* > .05).

**Table 1.** Brain-dead donor characteristics, grouped by DGF.

Variables	All donors (*n* = 102)	DGF (*n* = 44)^a^	non-DGF(*n* = 155)^a^	*p* value
Age, years, median (IQR)	47 (33–54)	48 (35–55)	47 (33–54)	.483
Sex, male, *n* (%)	84 (82)	33 (75)	131 (85)	.143
BMI, kg/m^2^, median (IQR)	22 (21–24)	23 (22–25)	23 (21–24)	.009
Extended‐criteria donors, *n* (%)	18 (18)	10 (23)	26 (17)	.365
Hypertension, *n* (%)	23 (23)	17 (39)	28 (18)	.004
Diabetes, *n* (%)	9 (9)	8 (18)	10 (7)	.032
Coronary artery disease, *n* (%)	0 (0)	0 (0)	0 (0)	n/a
Chronic kidney disease, *n* (%)	0 (0)	0 (0)	0 (0)	n/a
Proteinuri*a* ≥ 2+, *n* (%)	11 (11)	7 (16)	14 (9)	.263
Hepatitis C virus positive, *n* (%)	0 (0)	0 (0)	0 (0)	n/a
Active smoking, *n* (%)	42 (41)	21 (48)	62 (40)	.359
Causes of brain death, *n* (%)				.705
Trauma	47 (46)	18 (41)	75 (48)	–
Cerebral hemorrhage	43 (42)	19 (43)	64 (41)	–
Ischemic stroke	4 (4)	2 (5)	6 (4)	–
Cerebral anoxia	5 (5)	3 (7)	6 (4)	–
Other	3 (3)	2 (5)	4 (3)	–
Cardiac arrest before donation, *n* (%)	22 (22)	17 (39)	26 (17)	.002
Course of disease, d, median (IQR)	4 (2–8)	5 (3–9)	4 (2–8)	.172
Hospital’s stay, d, median (IQR)	3 (2–4)	3 (3–4)	3 (2–4)	.069
Infection, *n* (%)	48 (47)	26 (59)	70 (45)	.103
CRRT, *n* (%)	64 (63)	33 (75)	94 (61)	.080
AKI, *n* (%)	60 (59)	44 (100)	72 (47)	<.001
Stage 1	22 (22)	8 (18)	35 (23)	–
Stage 2	27 (26)	23 (52)	29 (19)	–
Stage 3	11 (11)	13 (30)	8 (5)	–
aNLR, median (IQR)	12.6 (8.4–19.2)	12.6 (6.0–20.0)	12.6 (8.5–19.1)	.655
pNLR, median (IQR)	10.0 (7.1–18.5)	10.9 (7.1–26.8)	9.8 (7.1–16.3)	.161
ΔNLR > 0, *n* (%)	37 (38)	24 (59)	48 (34)	.004
Interval between pNLR and aNLR detection, d, median (IQR)	2 (2–3)	2 (2–3)	2 (2–3)	.148

DGF: delayed graft function; IQR: interquartile range; BMI: body mass index; CRRT: continuous renal replacement therapy; AKI: Acute kidney injury; NLR: the ratio of absolute neutrophil count and absolute lymphocyte count.

aNLR, tested within 24 h before evaluating brain death; pNLR, tested within 6 h before organ procurement; ΔNLR = pNLR – aNLR.

^a^The number of recipients with DGF is 44 and without DGF is 155, and majority of 102 donors donated two kidneys.

**Table 2.** Recipient characteristics according to DGF status.

Variable	All (*n* = 199)	DGF (*n* = 44)	Non-DGF (*n* = 155)	*p* value
Age, years, median (IQR)	45 (34–54)	43 (33–55)	46 (35–54)	.872
Sex, male, *n* (%)	140 (70)	34 (77)	106 (68)	.255
BMI, kg/m^2^, median (IQR)	22 (19–24)	22 (19–26)	22 (19–24)	.216
Hypertension, *n* (%)	146 (73)	35 (80)	111 (72)	.293
Diabetes, *n* (%)	36 (18)	7 (16)	29 (19)	.670
Previous kidney transplant, *n* (%)	9 (5)	0 (0)	9 (6)	.211
Cause of ESRD, *n* (%)				.803
Diabetes	32 (16)	6 (14)	26 (17)	–
Hypertension	53 (27)	12 (27)	41 (27)	–
Polycystic kidney disease	8 (4)	3 (7)	5 (3)	–
Glomerulonephritis	32 (16)	6 (14)	26 (17)	–
Other or unknown	74 (37)	17 (39)	57 (37)	–
Duration of dialysis before transplant, months, median (IQR)	7 (3–12)	8 (4–17)	7 (2–12)	.713
Female kidney implanted into males, *n* (%)	19 (10)	7 (16)	12 (8)	.142
Panel reactive antibody (+), *n* (%)	6 (3)	2 (5)	4 (3)	.616
Blood type, *n* (%)				.383
A	56 (28)	17 (39)	39 (25)	–
B	53 (27)	10 (23)	43 (28)	–
AB	14 (7)	3 (7)	11 (7)	–
O	76 (38)	14 (32)	62 (40)	–
Cold ischemia time, h, median (IQR)	6 (3–7)	6 (3–8)	6 (3–7)	.629

ESRD: end stage renal disease.

**Figure 2.**
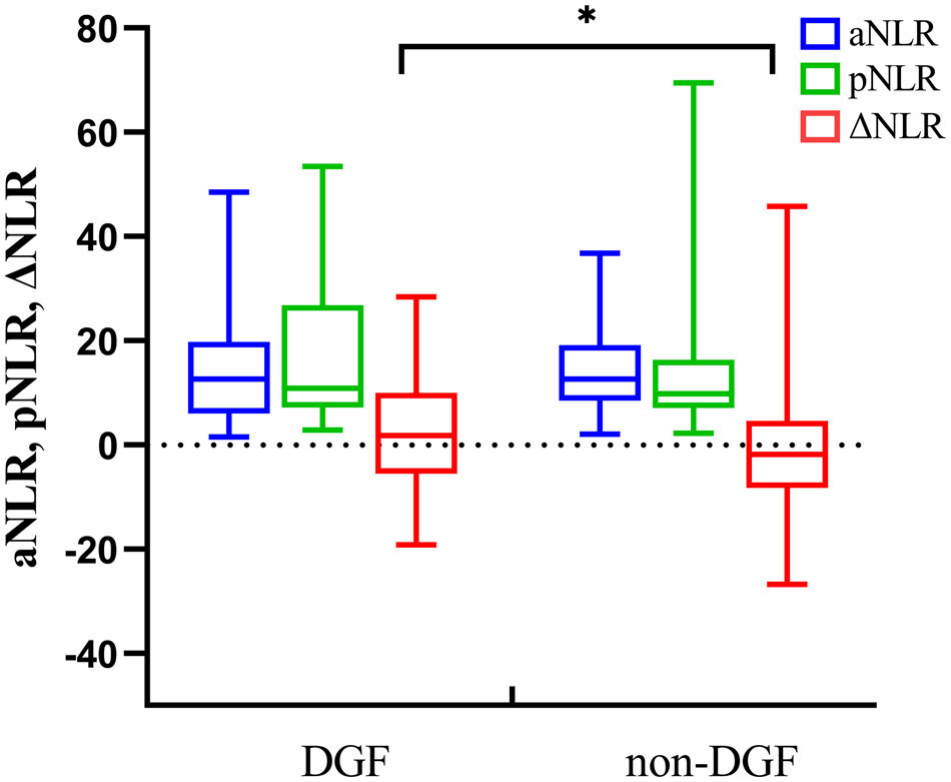
Dynamic change of aNLR, pNLR, and ΔNLR between DGF group and non-DGF group. aNLR, tested within 24 hours before evaluating brain death; pNLR, tested within 6 h before organ procurement; ΔNLR = pNLR – aNLR. *p < .05.

The median NLR value within 24 h before evaluating brain death were similar between the DGF (12.6, 95% CI 6.0 − 20.0) and non-DGF group (12.6, 95% CI 8.5 − 19.1; *p* = .655). The median preoperative NLR value in the DGF group (10.9, 95% CI 7.1 − 26.8) seemed higher over the non-DGF group (9.8, 95% CI 7.1 − 16.3), but the difference was of no statistical significance (*p* = .161) (Table 1). Comparing with the non-DGF group, the proportion of increasing donor NLR (ΔNLR > 0) in the DGF group was significantly higher (59% vs 34%; *p* = .004) (Table 1 and Figure 2), while the interval between pNLR and aNLR detection was no significant difference between two groups (*p* = .148).

**Figure 3.**
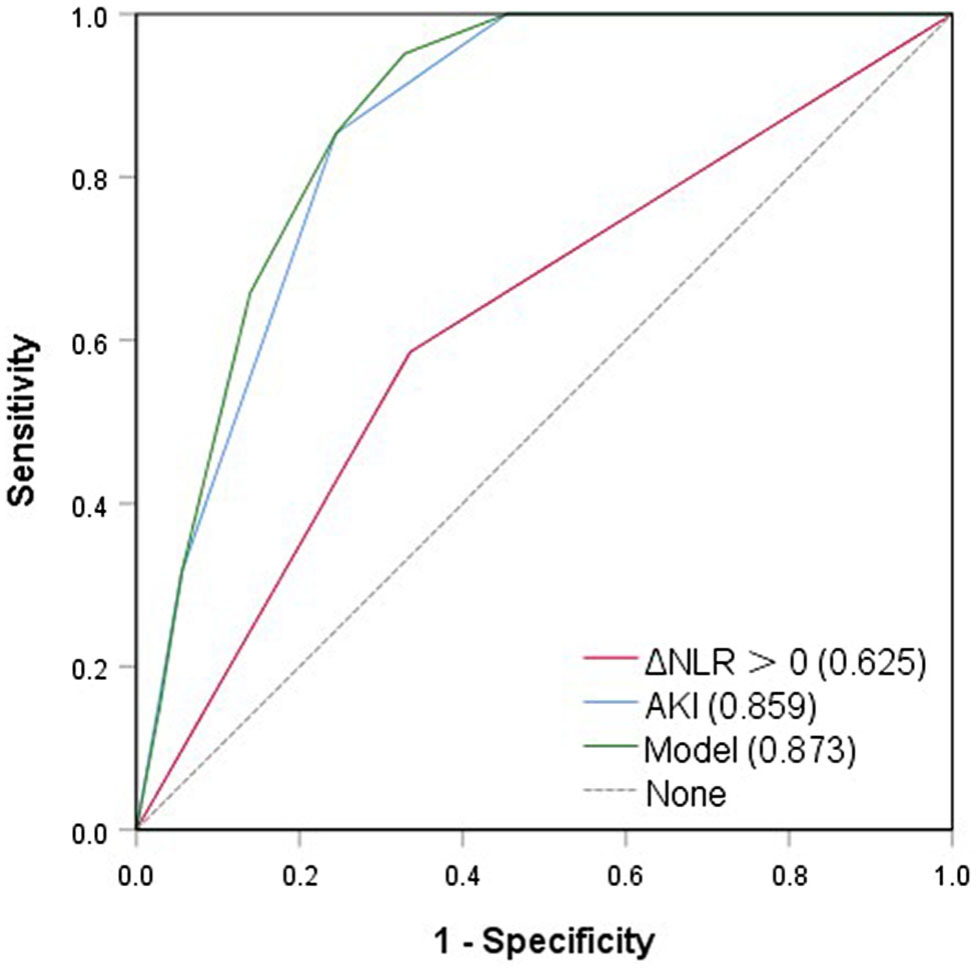
ROC curves of increasing NLR (ΔNLR > 0), AKI and the model including increasing NLR and AKI in donors for the prediction of recipient DGF.

Twenty‐two percent of recipients following renal transplantation developed DGF. Univariate and multivariate analysis were performed to analyze associated factors with DGF. In univariate analysis, increasing NLR in donors was significantly associated with DGF (OR 2.8, 95% CI 1.2 − 6.6; *p* = .018). Besides, other risk factors for DGF related to brain-dead donors included higher BMI (*p* = .018), hypertension (*p* = .014), diabetes (*p* = .032), occurrence of cardiac arrest (*p* = .006), and AKI (*p* < .001). Multivariate model 1 showed increasing donor NLR remained to be related to posttransplant DGF (OR 2.6, 95% CI 1.0 − 6.4; *p* = .040) after adjustment of BMI, hypertension, diabetes, and the occurrence of cardiac arrest. However, in another model that included donor AKI, increasing donor NLR lost its significant association with the risk of posttransplant DGF (OR 2.1, 95% CI 0.7 − 6.4; *p* = .174), while AKI in brain-dead donors was strongly related to the post-transplant DGF (OR 4.5, 95% CI 2.7 − 7.6; *p* < .001) (Table 3). The results of ROC curve analysis in predicting DGF for increasing donor NLR, AKI and the combination of increasing NLR and AKI in donors are shown in Figure 3. The AUC values of increasing NLR and AKI in donors were 0.625 (95% CI 0.526–0.723) and 0.859 (95% CI 0.806–0.912), respectively. The AUC value of the model including increasing NLR and AKI in donors increased to 0.873 (95% CI 0.823–0.923). Besides, the AUC value of increasing donor NLR was superior to other possible markers of DGF like donor age, cold ischemia time, and female kidney implanted into males (Supplementary File 1).

**Table 3.** Univariate and multivariate analysis of factors affecting DGF using GEEs.

	Unadjusted OR		Adjusted OR^a^		Adjusted OR^b^	
	OR (95% CI)	*p* value	OR (95% CI)	*p* value	OR (95% CI)	*p* value
BMI, kg/m^2^	1.2 (1.0 − 1.3)	.018	1.2 (1.0–1.4）	.023	1.0 (0.9–1.2)	.866
Hypertension	2.8 (1.2–6.3)	.014	2.0 (0.7–5.8)	.200	0.9 (0.3–3.5)	.933
Diabetes	3.2 (1.1–9.4)	.032	2.4 (0.5–10.9)	.249	2.9 (0.9-9.6)	.082
Cardiac arrest before donation	3.2 (1.4–7.5)	.006	2.9 (1.0–7.9)	.044	2.8 (0.9–8.4)	.074
Acute kidney injury	4.4 (2.8–7.0)	<.001	–	–	4.5 (2.7–7.6)	<.001
ΔNLR > 0	2.8 (1.2–6.6)	.018	2.6 (1.0–6.4）	.040	2.1 (0.7–6.4)	.174

OR: odds ratio; CI: confidence interval.

^a^Variables included in the multivariable model included BMI, hypertension, diabetes, and occurrence of cardiac arrest.

^b^Variables included in the multivariable model included BMI, hypertension, diabetes, occurrence of cardiac arrest and AKI.

## Discussion

This study explored the predictive value of dynamic changes of donor NLR in graft DGF. The dynamic changes of donor NLR have a significantly predictable role in the early graft outcome. Although AKI in donors was strongly related to the development of DGF in recipients, the combination with increasing donor NLR would further improve its predictive value.

One of the multiple risk factors in relation to the incidence of DGF after kidney transplantation are donor-related factors. A study reported kidneys procured from brain-dead donors were at higher rate of DGF [27]. In our study, the incidence of DGF in recipients was 22%, similar to other studies [25,28]. As is known, the pathophysiology of brain death exerts detrimental effects on organ function. Excessive secretion of catecholamines surrounding brain death increase systemic vascular resistance, leading ischemia and hypoxia in organs. With the following decline of catecholamine levels, the vascular resistance decreases but a cardiovascular collapse is presented [29,30]. The hemodynamic instability induces ischemia/reperfusion injury on kidney, damaging extensive endothelial cells and promoting a release of inflammatory cytokines [20]. Additionally, the hormonal and metabolic changes during brain death aggravate systemic and local inflammatory response [31]. A large number of inflammatory cytokines triggered by brain death were detected in previous experimental and clinical studies [32–34]. The activated inflammatory system may cause graft dysfunction in the recipients [35].

NLR is a simple and inexpensive parameter indicating inflammation state. It integrates general information of three systems: vegetative nervous system, neuroendocrine and immune systems [36]. Some studies have reported that a correlation between high NLR and development of DGF or acute rejection in the recipients after kidney transplantation [16,18,37]. In these studies, the study population were recipients and NLR value was calculated at a single time point. Moreover, the cutoff value for NLR predicting graft outcome remains inconsistent. Actually, organs before harvesting have been injured by a series of events from severe brain damage to brain death. The pathophysiological change as well as the inflammatory status surrounding brain-dead donor is dynamic. NLR is a dynamic marker with a fast response to insults and suggests improvement or deterioration of the clinical status [36]. In this study, we hypothesize that the dynamic trend of NLR in brain-dead donors may provide insight into the inflammatory status of kidney. To our knowledge, this are few studies exploring the utility of dynamic change of brain-dead donor NLR in DGF prediction.

In our study, despite the NLR value before organ procurement in the DGF group is higher than non-DGF group, this result is of no statistical difference. In univariate analysis, increasing donor NLR was proved to correlate with the risk of post-transplant DGF. When adjusted by BMI, hypertension, diabetes, and occurrence of cardiac arrest, increasing donor NLR was still associated with posttransplant DGF. But this relation lost statistical significance when adjusted by the aforementioned factors and donor AKI. Donor AKI become the only risk factor related to the posttransplant DGF. Notably, recipients in the DGF group all received donations from donors with AKI. We found that increasing donor NLR was independently associated with donor AKI (Supplementary File 2). We speculate that donor AKI may be the most significant factor related to the posttransplant DGF and the role of increasing donor NLR in predicting DGF may be limited owing to the small sample size. Alternatively, there may exist some other unexpected or confounding factors related with graft dysfunction.

Additionally, donor BMI was observed related to the occurrence of DGF in this study. Some authors presumed that it might result from the impact of immune system in the overweight/obese donors and the insufficient cooling of organs taken from these donors. Because the perirenal fat is not always removed adequately [38,39]. We speculate that the increase in BMI, as a metabolic and systemic disease, may be relevant with DGF.

Early prediction of graft dysfunction could serve as a warning for clinicians to initiate renal protective measures. Various factors in brain-dead donors have been proposed for predicting the development of DGF in recent years. Similar to some studies [6,40–42], donor AKI is related to the occurrence of DGF in this study. As mentioned earlier, the hemodynamic instability following brain death induces ischemic/reperfusion injury on peripheral organs. Massive pro-inflammatory factors impair kidney function and cause AKI [30]. The development of DGF mainly results from an ischemic injury to the kidneys exacerbated by reperfusion syndrome. Thus, donation from donors with AKI could influence the clinical graft outcome, facilitating clinicians to identify possible renal graft dysfunction.

Our study also has limitations. First, it is a small-sized study of a single center which inevitably introduces biases in data collection. The validation from a large multi-institutional study is needed. Second, despite adjustment for some critical factors, there may still exist unmeasured confounders given the observational study design. Nevertheless, it has strength with regard to homogeneity of patient population, donor management, standard immunosuppression, and other postoperative management protocols in a single center.

## Conclusion

Dynamic changes of NLR in brain-dead donors were promising in predicting the development of DGF in recipients. Furthermore, increasing NLR combining AKI in brain-dead donors had significantly better predictive efficacy and may assist in early recognition and management of renal graft dysfunction. A large and prospective study is warranted to validate this relationship in the future.

## Supplementary Material

Supplemental Material

Supplemental Material
