# Supplementary material for: Dynamic changes of neutrophil-to-lymphocyte ratio in brain-dead donors and delayed graft function in kidney transplant recipients
Source: Ren Fail. 2022 Nov 8;44(1):1897–903. doi: 10.1080/0886022X.2022.2141646 (PMC9648373; doi:10.1080/0886022X.2022.2141646)
Supplement: Supplemental Material [file IRNF_A_2141646_SM7359.pdf]

Supplementary materials

Table 1 Brain-dead donor characteristics, stratified by acute kidney injury

Variable	All donors (N = 102)	AKI (n = 60)	non-AKI (n = 42)	*p* value
Age, y, median (IQR)	47 (33-54)	49 (39-55)	42 (31-52)	0.076
Sex, male, n (%)	84 (82)	51 (85)	33 (79)	0.402
BMI, kg/m2, median (IQR)	22 (21-24)	23 (22-25)	22 (20-23)	0.003
Extended-criteria donors, n (%)	18 (18)	15 (25)	3 (7)	0.020
Hypertension, n (%)	23 (23)	18 (30)	5 (12)	0.031
Diabetes, n (%)	9 (9)	6 (10)	3 (7)	0.884
Coronary artery disease, n (%)	0 (0)	0 (0)	0 (0)	n/a
Chronic kidney disease, n (%)	0 (0)	0 (0)	0 (0)	n/a
Proteinuria ≥2+, n (%)	11 (11)	7 (12)	4 (10)	0.985
Hepatitis C virus positive, n (%)	0 (0)	0 (0)	0 (0)	n/a
Active smoking, n (%)	42 (41)	28 (47)	14 (33)	0.178
Causes of death, n (%)				0.823
Trauma	47 (46)	25 (42)	22 (52)	
Cerebral hemorrhage	43 (42)	28 (47)	15 (36)	
Ischemic stroke	4 (4)	2 (3)	2 (5)	
Cerebral anoxia	5 (5)	3 (5)	2 (5)	
Other	3 (3)	2 (3)	1 (2)	
Cardiac arrest before donation, n (%)	22 (22)	15 (25)	7 (17)	0.314
Course of disease, d, median (IQR)	4 (2-8)	4 (2-7)	4 (2-9)	0.829
Infection, n (%)	48 (47)	27 (45)	21 (50)	0.619
aNLR, median (IQR)	12.6 (8.4-19.2)	12.3 (7.1-17.9)	13.8 (9.2-20.2)	0.175
pNLR, median (IQR)	10.0 (7.1-18.5)	7.9 (11.8-23.1)	8.7 (6.2-13.0)	0.013
ΔNLR > 0, n (%)	37 (37.8)	27 (46.6)	10 (25.0)	0.031

BMI, body mass index; AKI, Acute kidney injury; NLR, the ratio of absolute neutrophil count and absolute lymphocyte count; aNLR, tested within 24 hours before evaluating brain death; pNLR, tested within 6 hours before organ procurement; ΔNLR = pNLR – aNLR; n/a, not applicable.

Table 2 Univariate and multivariate logistic analysis of factors associated with donor acute kidney injury

	Unadjusted OR		Adjusted OR[a]	
	OR (95% CI)	*p* value	OR (95% CI)	*p* value
BMI, kg/m2	1.3 (1.1-1.5)	0.007	1.2 (1.0-1.5)	0.020
Extended - criteria donors	4.3 (1.2-16.1)	0.028	6.4 (1.6-25.6)	0.009
Hypertension	0.3 (0.1-0.9)	0.037	-	-
pNLR	1.0 (1.0-1.1)	0.080	-	-
Δ NLR > 0	2.6 (1.1-6.3)	0.033	2.8 (1.1-7.4)	0.030

[a] adjusted by variables including BMI, Extended-criteria donors, hypertension, and Δ NLR > 0 .
