# Supplementary material for: Dynamic changes of neutrophil-to-lymphocyte ratio in brain-dead donors and delayed graft function in kidney transplant recipients
Source: Ren Fail. 2022 Nov 8;44(1):1897–903. doi: 10.1080/0886022X.2022.2141646 (PMC9648373; doi:10.1080/0886022X.2022.2141646)
Supplement: Supplemental Material [file IRNF_A_2141646_SM7357.pdf]

Supplementary materials

Table 1 Comparisons of the Areas Under the Curve (AUC) values among the parameters of predicting DGF

Parameters	AUC	95%CI	*p* value
Cold ischemia time	0.523	0.425-0.621	0.637
Donor age	0.535	0.439-0.631	0.483
Female kidney implanted into males	0.541	0.441-0.641	0.409
ΔNLR > 0	0.625	0.526-0.723	0.015
AKI	0.859	0.806-0.912	< 0.001
ΔNLR > 0 and AKI	0.873	0.823-0.923	< 0.001

AKI, acute kidney injury; DGF, delayed kidney function; aNLR, tested within 24 hours before evaluating brain death; pNLR, tested within 6 hours before organ procurement; ΔNLR = pNLR – aNLR.
